# POCUS for Diastolic Dysfunction: A Review of the Literature

**DOI:** 10.24908/pocus.v8i1.15803

**Published:** 2023-04-26

**Authors:** Samantha A King, Alexis Salerno, Jessica V Downing, Zachary R Wynne, Jordan T Parker, Taylor E Miller, Semhar Z Tewelde

**Affiliations:** 1 Department of Emergency Medicine, University of Maryland School of Medicine Baltimore, MD USA; 2 Program in Trauma/Surgical Critical Care, The R Adams Cowley Shock Trauma Center, University of Maryland Medical Center Baltimore, MD USA; 3 Department of Emergency Medicine, University of Maryland Medical Center Baltimore, MD USA

**Keywords:** echocardiography, Point of Care Ultrasound, diastology, HFpEF

## Abstract

Emergency and critical care physicians frequently encounter patients presenting with dyspnea and normal left ventricular systolic function who may benefit from early diastolic evaluation to determine acute patient management. The current American Society of Echocardiography Guidelines approach to diastolic evaluation is often impractical for point of care ultrasound (POCUS) evaluation, and few studies have evaluated the potential use of a simplified approach. This article reviews the literature on the use of a simplified diastolic evaluation to assist in determining acute patient management.

## Introduction

Symptomatic heart failure continues to have a significant associated morbidity and mortality^1^. A portion of patients hospitalized for symptomatic heart failure will be found to have preserved left ventricular systolic function (HFpEF) 2. There are various causes of HFpEF, but most commonly patients will be diagnosed with diastolic dysfunction 1. 

In 2016, the American Society of Echocardiography (ASE) and the European Association of Cardiovascular Imaging (EACI) published guidelines for the evaluation of diastolic dysfunction using echocardiography 3. These guidelines are based on expert consensus and recommend multiple 2D and Doppler measurements to help diagnose diastolic dysfunction within the correct clinical context. Many physicians have found it impractical in the emergency department and critical care setting to collect all measurements included in the ASE guidelines at the bedside. This difficulty may be due to multiple factors 4. For example, if the patient has hypoxemia or dyspnea, they may be unable to lay flat or be properly positioned for the sonographer to obtain an adequate apical 4 chamber view. Furthermore, many emergency physicians do not have the advanced training in echocardiography to perform these measurements. 

In this article, we review the literature for evaluation of diastolic dysfunction using a simplified echocardiographic approach.

## Methods

We reviewed the literature to look for simplified approaches to evaluating diastolic dysfunction using echocardiography. We identified articles through a systematic search of the Medline (PubMed) and EMBase databases for relevant articles published after September 1, 2016. Our search terms were 'diastolic heart failure'/exp OR 'diastolic function'/exp) AND ('transthoracic echocardiography'/exp OR 'point of care ultrasound'/exp) for EMBase and (("Heart Failure, Diastolic"[Mesh] OR "Diastole/physiology"[Mesh]) AND ("Echocardiography"[Mesh] OR "Ultrasonography"[Mesh])) for PubMed.

## Results

**Figure 1:**
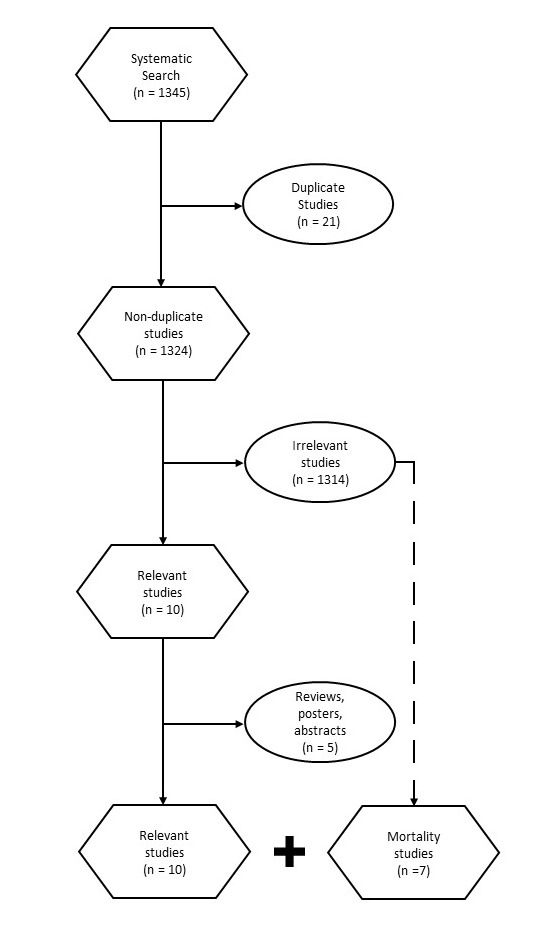
Flow diagram of studies selected for review.

We identified 1345 studies satisfying the search terms (Figure 1). We excluded 21 duplicate studies and 1314 irrelevant studies. We excluded studies using transesophageal echocardiography, 3D echo-cardiography or volumetric strain echocardiography measurements. We also excluded studies which included patients with decreased left ventricular ejection fraction, pediatrics, congenital or valvular heart disease. Lastly, we excluded review articles, posters, and abstracts. We found five articles responsive to our initial question. In addition, we found seven articles which discussed echocardiographic identifiers of mortality rather than diagnostic signs of diastolic dysfunction. The authors felt this was important to the overall topic and should be included in this report. 

The characteristics of the included studies are summarized in Table 1 and Table 2. Most of the studies used retrospective data. Seven studies enrolled inpatients and three enrolled outpatient clinic patients. The sonographers for these studies were usually not identified in the reports, but we assumed they were certified cardiology echocardiographers with interpretation by a cardiologist if not otherwise stated. Most of the studies did not reference the 2016 ASE guidelines or compare the data directly to these guidelines.

**Table 1:** Studies discussing the diagnosis of diastolic dysfunction using a simplifiedmethods

Year	Author	Journal title	Location	Setting	Article type	# of patients	Clinical question	Diastolic method used	Outcome
2016	Ballo	Int J Cardiol	Italy	Primary care	Feasibility; Correspondence	885	Evaluate agreement of sDD and cDD	E:A, E <160ms, E/e’ >10	Fair concordance; sDD underestimate severity of DD
2017	Lekavich	J Card Fail	NC, USA	Inpatient cases with outpatient controls	Case matched	310	Detect echo markers associated with incidence of HFpEF	cDD	LA diam and Ees changes correlate with HFpEF
2018	Del Rios	Crit Ultrasound J	Il, USA	ED	2° analysis of prospective data	48	Ascertain inter-rater agreement in sDD between EP and cards	e’_A_ <9 cm/s	Good agreement between EP and cardiologist interpretation
2018	Reddy	Circulation	MI, USA	Outpatient clinic	Retrospective	414	Develop diagnostic scoring system for HFpEF	Obtained all echo markers	E/e’ >9, PASP > 35mmHg correlated with HF. H2FPEF score
2019	Mielnicki	Anaesthesiol Intensive Ther	Poland	Intensive care unit	Observational	200	Analyze utility of systolic TD for diagnosis of DD	Obtained all echo markers for cDD	Velocity of systolic TD is statistically lower with DD
sDD indicates simplified diastolic evaluation; cDD:,comprehensive diastolic evaluation; Ees, ventricular elastance; EP, emergency physician; HfpEF, heart failure with preserved ejection fraction; PASP, pulmonary artery systolic pressure; HF, heart failure; and TD, tissue Doppler.									

**Table 2:** Studies discussing the mortality associated with diagnosis of diastolic dysfunction

Year	Author	Journal Title	Location	Setting	Article Type	# of Patients	Clinical Question	Outcome
2016	Hsiao	PLoS One	Taiwan	HF Clinic	Prospective	162	Evaluate LAEI as predictor for progression to HF	LAEI is good predictor of event rate with DD
2016	Kim	Int J Med Sci	South Korea	Inpatient surgery	Prospective	242	Relation of echo parameters & DD on post renal transplant CV complications	↑ E/e’ ratios predict MACE In post kidney transplant patients
2017	Issa	J Cardiovasc Echo	FL, USA	Inpatient	Retrospective cross-sectional	80	Identify echo markers that correlate with need for HF hospitalization	LAmVI correlated strongly with need for HF hospitalization
2017	Johansen	Am Heart J	Denmark		Longitudinal cohort	1851	Prognostic value of traditional echo measures, establish a new grading DDF	Abnormal e’ ↑ risk of MACE
2020	Higashi	J Anesth	Japan	Inpatient	Retrospective	965	Incidence of post-op HFrelated to pre-op E/e’	E/e’ (≥15) associated w/ increase postop HF
2020	Oike	Int J Cardiol	Japan	Inpatient	Prospective	448	Evaluate relationship of a’ to CV events in pts with HFpEF	a’ <7.5 cm/s ↑ CV event rate
2020	Hoshida	Open Heart	Japan	Inpatient	Prospective	552	Ed/Ea in relation to all-cause mortality in HFpEF	Ed/Ea increased correlation with mortality is only related for short term after hospitalization
LAEI indicates left atrial expansion index; MACE, major adverse cardiac event; LamVI, left atrial minimum volume index; HF, heart failure; HfpEF, heart failure with preserved ejection fraction; CV, cardiovascular; Ed, diastolic elastance; and Ea, arterial elastance.								

## Discussion

Recognizing echocardiographic evidence of diastolic dysfunction can be very helpful when attempting to differentiate the underlying etiology of patients presenting with dyspnea and it has also been found useful as a clinical predictor for patients with sepsis without the need for invasive measurements 4, 5, 6. 

In 2016, the ASE and the EACI published an updated guideline for the evaluation of diastolic dysfunction. The goal of the update was to create a more simplified set of guidelines, as it was noted that the prior guidelines, published in 2009, were considered too complex and led to difficulty for providers in diagnosing diastolic dysfunction 3. The updated guidelines review the literature and set forth steps for diagnosis based on the ejection fraction (EF) of the patient and other factors, such as concerns for constrictive or restrictive pericarditis.

For patients found to have a normal LVEF, the guidelines recommend the evaluation of four parameters: E/E’ ratio, septal e’ or lateral e’ velocity, tricuspid regurgitation (TR) velocity and left atrial (LA) volume index. The first three values are to be obtained through the apical 4 chamber (A4C) view with pulse wave Doppler of the mitral valve flow, tissue Doppler imaging of the mitral valve septum or lateral plane, and continuous wave Doppler of the tricuspid valve. The last calculation, volumetric analysis of the left atrium, requires a volume assessment in the traditional A4C and then an additional orthogonal view from the A4C view. For diastolic dysfunction to be diagnosed, three or four of these measurements must be abnormal. If only one or none of these values is noted to be abnormal, diastolic dysfunction is excluded. If two of these values are noted to be abnormal, then the result is “indeterminate.” Unlike the 2009 guidelines, the 2016 guidelines do not specify further gradation of diastolic dysfunction in patients with normal EF.

The 2016 ASE guidelines, despite simplifying the 2009 guidelines, still present challenges for a timely evaluation at bedside in the ED setting. One of the biggest challenges is that providers in the emergency department are trained in POCUS. POCUS is a limited survey seeking to answer a simple yes or no question. However, the diagnosis of diastolic dysfunction often requires more complicated parameters and measurements than what POCUS can provide involving both echogenic and clinical evaluation.

A review by Greenstein highlights the difficulties in the critical care setting, many of which also apply in the ED setting 4. Current guidelines require the assessment of both mitral and tricuspid flow patterns, with the latter potentially difficult to obtain a crisp Doppler signal. It also requires both septal and lateral measurements of the mitral valve annular velocity without clarifying their added benefit over a single plane measurement. If these views can be obtained, they may provide a sufficient basis for a diagnosis or exclusion of diastolic dysfunction. However, if these views cannot be obtained or are not sufficient for diagnosis, then the guidelines call for obtaining potential LA volumetric assessment via two orthogonal views. This measurement can be time-consuming and is not a measurement typically performed by providers trained in POCUS. While the first view may be obtained in the A4C view to obtain this measurement, a second orthogonal view may be more difficult to obtain. Additionally, the ability to obtain an A4C view, orthogonal views, or multiple views of the same area is likely to be limited by patient positioning, presence of monitors, and other external devices. It may be impractical if not impossible to turn or reposition a critically ill patient to provide optimal evaluation. All these factors make the current diastolic function assessment by ASE guidelines impractical in the acute care setting.

In our review there was substantial heterogeneity across studies in the evaluation of diastolic dysfunction and the markers of mortality. Most of the studies looked at mitral valve Doppler velocities 7, 8, 9, 10, 11, while a few looked at left atrial volumes 12, 13, 14. Four of the articles looked at the ratio of E/e’ and found an associated increase in mortality with those patients who had an increase in the E/e’ value 10, 11. These findings are similar to those presented in other articles published prior to the 2016 guidelines. In 2016, Lanspa et al looked at septic ICU patients and found that the E/A ratio, e’ velocity and E/e’ ratio were the most important in predicting 28-day mortality 15. However, in our review of the literature, the values of E/e’ and e’ for diagnosing diastolic dysfunction varied among the different studies and ranged from E/e’ >9 to E/e’ >15 4, 7, 8, 11, 16, 17. One article from 2018 discussed the development of a diagnostic scoring system for diagnosing HfpEF 8. It is the only article we found that combined echocardiographic measurements with clinical variables such as body mass index, hypertension, atrial fibrillation, pulmonary hypertension, and age. The results of the study showed robust performance in their 100-patient cohort 8. Additionally, each study had varying inclusive and exclusive criteria including presence of dysrhythmia, valvular pathology, which may limit the general applicability.

Two studies directly compared a simplified method with a comprehensive method of evaluating for diastolic dysfunction 7, 16. The Ballo et al correspondence reported that their simplified method using mitral valve Doppler values had a fair concordance with the comprehensive evaluation and often underestimated the severity of diastolic dysfunction 7. This is in contrast to Del Rios et al, which showed good agreement between emergency physicians (EPs) and cardiologists using only the average e’ value 16. This is also in contrast to Lanspa et al (written prior to 2016 and not included in our review), which showed overestimation of the severity of diastolic dysfunction using the simplified approach as compared to the 2009 guidelines 15. Ultimately, a simplified approach to diastolic evaluation can help better risk stratify patients presenting with dyspnea. While it does not preclude the need for further evaluation of diastolic dysfunction, a simplified evaluation can provider further information within the initial evaluation and treatment.

## Limitations

While a simplified diastolic analysis can provide further diagnostic information, the technique has some drawbacks. If not properly obtained, these measurements would likely have more limited accuracy 18. In addition, other conditions a patient may have (e.g., restrictive pericarditis, atrial fibrillation, or heart failure) can impact the accuracy of the tissue Doppler measurement if they are unrecognized, and this could lead to a false interpretation 18. Finally, if measurements are not reviewed in the context of the clinical picture, one risks a diagnosis of non-clinically relevant diastolic dysfunction 18. However, studies have shown that EPs are capable of identifying clinically significant diastolic dysfunction, and other studies have shown that non-cardiologist have good inter-rater reliability for the assessment of diastolic dysfunction 19, 20, 21, 22, 23, 24. 

The accuracy and utility of a simplified evaluation method for diastolic dysfunction likely needs further investigation and validation studies. There are a few proposals for a simplified diastolic dysfunction evaluation, but robust research in this area, including prospective trials, is lacking. Much existing research in this area is based on retrospective review of comprehensive echocardiographic examinations with parameters that are increased in patients who have been diagnosed with diastolic dysfunction. Furthermore, a few of the articles in our review proposed a simplified diagnostic approach, yet still required complex calculations for the evaluation of diastolic dysfunction.

Simplified diastolic evaluation methods do not preclude the need for a comprehensive echocardiogram or provide a definitive diagnosis, but it helps physicians target their care in a timely fashion when comprehensive echocardiography is neither feasible nor readily available.

## Conflicts of Interest

The authors have no conflicts of interest to disclose.
